# Thanks

**Published:** 1889-02

**Authors:** 


### THANKS.

We desire to acknowledge our indebtedness to Dr. W. D. Miller, Berlin, for his earnest efforts in behalf of the journal, and his most efficient aid in securing the corps of foreign correspondents. Without his kindly assistance, we do not see how we should have succeeded in organizing our foreign staff as well as we have. The amount of labor necessary to set in motion and get to running smoothly an enterprise of the magnitude of the International Dental Journal is only to be appreciated by those who have gone through with it.

Mankind is not altogether sordid and selfish, notwithstanding such is the impression obtained from contact with the business world in general. The above reflection was called forth by an unasked favor that was granted the INTERNATIONAL DENTAL JOURNAL COMPANY by the Welch Dental Manuf'g Co. by allowing us the use of their mailing list, in sending out our twenty thousand circular letters to the profession, we take great pleasure in publicly acknowledging their generosity. There is probably no larger or more accurate mailing list extant at the present time than theirs. It costs a great amount of labor and money to keep a corrected mailing list of the dentists in this and foreign countries, and this enterprising house has spared no pains to be up to the mark in this direction. Knowing the full value of printer's ink they have kept themselves well before the profession.

We are in receipt of a neat little appointment book published by R. I. Pearson & Co., also a kindly letter asking for a bill for the copies of the INTERNATIONAL sent, as we supposed, in exchange for copies of the *Western Dental Journal* published by that firm. Their letter shows such a broad and praise-worthy spirit that we take the liberty of publishing it.

KANSAS CITY, Dec. 31st, 1888.

DR. W. X. SUDDUTH,

*Dear Sir*:—Please send INTERNATIONAL DENTAL JOURNAL for 1889, as follows, * * * * * and bill to us. Your Journal being largely dependent upon the profession for support, we have not heretofore asked it sent to the Editors of the *Western Dental Journal* as complimentary or in exchange; but have always paid Dr. Barrett for the copies, and we prefer to continue to do so Upon receipt of bill we will remit as usual, and may by that time have additional names.

(Signed), Yours truly,

R. I. PEARSON & CO.

Favors of like character, and the congratulatory and commendatory letters that we are continually receiving from the leading dealers and manufacturers of dental supplies, are the very best evidence that the INTERNATIONAL is appreciated by the business portion of the dental world. Our very rapidly increasing subscription list also gives positive evidence of its appreciation by the profession. So we say thanks to the dealers—thanks to the manufacturers—thanks to the profession, thanks all 'round.

